# Surgical techniques for facilitating laparoscopic intracorporeal orthotopic neobladder: initial experience

**DOI:** 10.1590/S1677-5538.IBJU.2017.0505

**Published:** 2018

**Authors:** Lianchao Jin, Mingshuai Wang, Feiya Yang, Yinong Niu, Nianzeng Xing

**Affiliations:** 1 Department of Urology, Beijing Chao-yang Hospital, Capital Medical University, Beijing, China; 2 Department of Urology, Peking University Shougang Hospital, Beijing, China

**Keywords:** Laparoscopy, Quality of Life, Cystectomy

## Abstract

**Purpose:**

To describe our technique and outcomes for laparoscopic intracorporeal ileal neobladder (ICNB) reconstruction.

**Materials and Methods:**

From April 2014 to November 2016, 21 patients underwent laparoscopic ICNB at our tertiary referral centre. ICNB with bilateral isoperistaltic afferent limbs and several technique improvements were introduced. Demographics, clinical, and pathological data were collected. Perioperative, 1-year oncologic, 1-year Quality of life and 1-year functional outcomes were reported.

**Results:**

ICNB was successfully performed in all 21 patients without open conversion and transfusion. Mean operative time was 345.6±66.9 min, including 106±22 min for LRC and PLND and 204±46.4 min for ICNB, respectively. Mean established blood loss was 192±146 mL. The overall incidence of 90-d complication was 33.3%, while major complication occurred in 4.8%. One-year daytime and night-time continence rates were 85.7% and 57.1%, respectively. One patient died from myocardial infarction six months postoperatively, and two patients had lung metastasis five months and six months respectively.

**Conclusions:**

We described our experience of 3D LRC with a novel intracorporeal orthotopic ileal neobladder, and the technique improvements facilitate the procedure. However, further studies are required to evaluate long-term outcomes of the intracorporeal neobladder with bilateral isoperistaltic afferent limbs.

## INTRODUCTION

Radical cystectomy (RC) with pelvic lymph node dissection (PLND) has become the standard treatment option for muscle-invasive and high-risk superficial bladder cancer. Laparoscopic or robotic- -assisted radical cystectomy is an alternative to open radical cystectomy with comparable oncological outcomes in multiple centers (1, 2). Following minimally invasive radical cystectomy, urinary diversion may be performed through an open approach or entirely within the abdomen (3, 4). For technical difficulties and relatively longer operative time, urologists were not optimistic about the intracorporeal neobladder (ICNB) formation at first (5). With the development of minimally invasive technique and device, ICNB was reconsidered in large medical centers in recent years (4, 6). To data, most of the intracorporeal urinary diversion was performed in robotic-assisted approach (7).

After Gill et al. firstly reported laparoscopic radical cystectomy (LRC) and ICNB in 2002 (3), laparoscopic intracorporeal U shape orthotopic neobladder was proposed in many centers (8-10). They simplified the procedure of ICNB construction, however, the function of the U shape neobladder is controversial as it is not in a global shape, which may result in relatively small neobladder capacity and higher neobladder pressure.

Hence, we describe a time efficient method of ICNB reconstruction and a series of technique improvements to overcome challenges during the procedure.

## MATERIALS AND METHODS

We reviewed medical records of patients who underwent LRC with ICNB formation in our tertiary center from April 2014 to November 2016. The study was approved by the Institutional Review Board of Beijing Chao-yang Hospital, Capital Medical University (Protocol number 2014-R-141), and all patients provided written informed consent. All surgeries were performed by one experienced surgeon (N.X.), who has over 200 cases experience of LRC with orthotopic ileal neobladder. Indications for LRC were muscle-invasive bladder cancer (T2-3, N0-x, M0), high risk and recurrent non-muscle-invasive cancer, T1G3, extensive non-muscle-invasive bladder cancer that could not be controlled by transurethral resection and intravesical therapy. Our exclusion criteria were tumor in the urethra, urinary incontinence, history of recurrent urethral strictures, abnormal abdominal straining, local radiotherapy and severe comorbidities and decompensated renal function.

Perioperative variables including patient's characteristics, surgical outcomes, postoperative complications, oncologic outcomes and Quality- -of-life data were analyzed. Postoperative complications were analyzed according to the Clavien-Dindo classification (11). After discharge, patients were followed up at 2 weeks, 3, 6, and 12 months, and then yearly. Neobladder function was assessed at 6 months postoperatively. Daytime or night-time continence referred to the requirement of <1 pad during daytime or night-time, respectively. Incontinence was defined as the need for more than 1 pad per day or night. Patients were asked to complete the European Organization for Research and Treatment of Cancer (EORTC) generic (QLQ-C30) and bladder cancer specific instruments (QLQ-BLM30) questionnaires for Quality of Life assessment every year.

### Surgical Techniques

**Figure 1:**
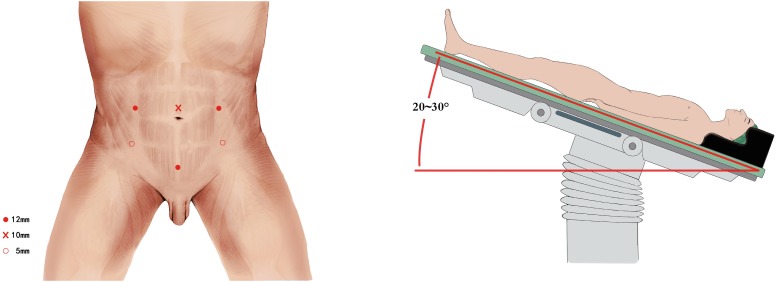
Trocar placement and patient position.

All procedures were performed using Olympus 3-D laparoscopic system. The patient was placed in a dorsal supine with a 30° Trendelenburg position. Six laparoscopic ports were utilized as shown in Figure-1. The first 10 mm port for the camera was placed 1 cm cephalad to the umbilicus in the midline. Two 12 mm ports were symmetrically placed at the level of the umbilicus on the left and right lateral to the rectus abdominis. Two 5 mm ports were placed 2-3 cm superior and medial to the anterior superior iliac spines on each side. The sixth 12 mm port was placed 1 cm cephalad to the pubic symphysis in the midline after finishing the LRC and PLND for Endo-GIA.

After the peritoneum was incised and the sigmoid colon was mobilized, the ureters were mobilized down to the bladder without injuring the periureteral vessels. Then, the umbilical arteries were dissected and divided between Hem- -o-lok clips. The peritoneum was incised along the vas deferens in the direction of the pouch of Douglas, and the seminal vesicles were completely dissected. The posterior layer of the Denonvilliers fascia was then incised and the pre-rectal fatty tissue could be visualized. The dissection was continued in a blunt and sharp fashion as far as possible towards the apex of the prostate. The bilateral bladder pedicles were divided between Hem-o-lok clips. Then, the Retzius space was dissected and the incision was continued in the lateral direction up to the external iliac vessels and the endopelvic fascia. After cleaning fatty tissue on the prostate, the endopelvic fascia was incised on both sides. Then, both puboprostatic ligaments were fully dissected, and the dorsal vein complex was ligated with 2-0 V-Loc suture. In nerve-sparing procedures, the bilateral prostatic fascia was dissected and the prostatic pedicles were clipped and cut with cold scissors step by step to detach neurovascular bundles from the prostatic capsule. In non-nerve-sparing procedures, LigaSure and ultrasonic scalpel were used to directly divide the prostatic pedicles towards the apex of the prostate. Then, the bilateral ureters were divided, and the urethra was clearly dissected and cut after clipped by Hem-o-lok. The cystoprostatectomy specimen was placed into an EndoCatch bag.

Extended PLND involved removal of nodal tissue cranially up to the inferior mesenteric artery, and including the internal iliac, presacral, obturator fossa and external iliac nodes was performed after the LRC. The lymph nodes were marked and put into the EndoCatch bag. Then, the bag was put in the abdomen and taken out after urinary diversion.

**Figure 2:**
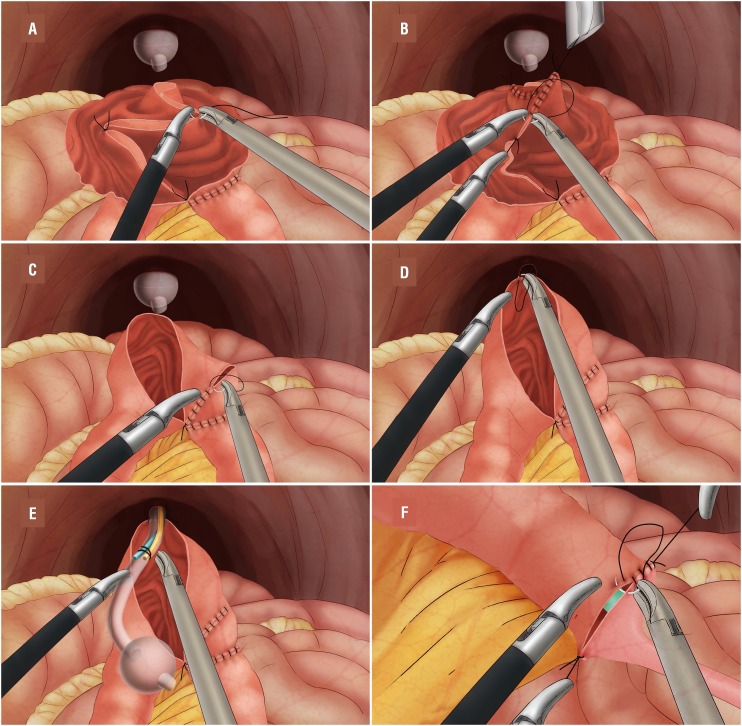
A) Interrupted suturing of the posterior wall of the reservoir using 2-0 Vicryl suture. B) Pulling the interrupted knots to tension ileum to facilitate suturing (“pulling technique”). C) Closure of the distal half of the anterior wall of the reservoir. D) The proximal half of the back wall of the reservoir was anastomosed with the urethra back wall. E) The catheter and two single J stents were introduced through urethra. F) The ureters were spatulated for 2-3 cm and were end-to-end anastomosed with ipsilateral limb in a continuous manner, using 3-0 vicryl sutures, respectively.

For intracorporeal orthotopic ileal neobladder construction, a 60 cm segment of ileum, 15-20 cm from the ileocecal junction, was isolated using laparoscopic Endo-GIA with a 60 mm staplers. The continuity of the small bowel was restored using the Endo-GIA with a 60 mm stapler, positioning the distal and proximal end of the ileum side to side with the antimesentery parts facing each other, and then the open end was closed with transverse firing of the Endo-GIA stapler. The proximal 10 cm segment of the isolated ileal segment was transected with ultrasonic scalpel and was manually anastomosed with the distal end in end-to-end manner. The middle 40 cm of the ileal segment was detubularised with scissors along its antimesenteric line, and the remaining 10 cm each side for reservoir limbs. The posterior wall of the reservoir was closed using 3-0 V-Loc suture in a simple continuous full thickness fashion. During the procedure, the posterior wall was firstly interrupted sutured using 2-0 Vicryl suture, and two assistants were pulling the interrupted knots to tension ileum to facilitate suturing (“pulling technique”) (Figures 2A and 2B). The distal half of the anterior wall of the reservoir was sutured using the 3-0 V-Loc suture (Figure-2C). The proximal half of the back wall of the reservoir was anastomosed with the urethra back wall, and the anterior wall of the reservoir was left open (Figure-2D). Then, the catheter and two single J stents were introduced from the opening through the urethra (Figure-2E). The opening was then anastomosed with the urethra and closed with 3-0 V-Loc suture using pulling technique. After the placement of a single J stent in the ureter and renal pelvis on each side, the ureters were spatula-ted for 2-3 cm and were end-to-end anastomosed with ipsilateral limb in a continuous manner using 3-0 Vicryl sutures, respectively (Figure-2F). The neobladder was then filled with 50 cc of saline to check for leakage. A drain was introduced and placed in the pelvis. The EndoCatch bag was retraced through an enlarged sixth port incision (usually 5 cm) in the midline of the abdominal wall for male, and the specimen was removed from vagina for female. The single J stents were removed two weeks after surgery, and the catheter was also removed two weeks after surgery after confirmed by cystography the absence of leakage.

## STATISTICAL METHODS

Mean values with standard deviations were computed and reported for continuous data. Median, range were used to report categorical data. Continuous and categorical variables were compared with Student t test and the X2 test, respectively. All p values <0.05 were considered statistically significant. Statistical analysis was performed using STATA v12 (StataCorp LLC, College Station, Texas, USA).

## RESULTS

LRC with ICNB was successfully accomplished in all 21 patients without open conversion. Tables 1 and 2 show the patient's characteristics and surgical outcomes. The mean age was 60 years old with a mean BMI 24.5 kg/m2. Of 21 patients, four patients received neoadjuvant chemotherapy and fourteen patients had history of transurethral resection of bladder tumor. The median Charlson comorbidity index score was 4, and ASA score was 2.

**Table 1:** Patient's Characteristics.

VARIABLES		RESULTS
Patients, n		21
Age (mean±SD), year		60±10.1
Male, n (%)		19 (90.5%)
BMI (mean ± SD), kg/m2		24.5±3.9
Neoadjuvant chemotherapy, n (%)		4 (19.0%)
Charlson comorbidity index (median [range])		4 (2-6)
**ASA score, n**		
	1-2	21
	3-4	0
Smoking history, n (%)		9 (42.9%)
Abdominal surgery history, n (%)		5 (23.8%)

**BMI** = body mass index; **ASA** = American Society of Anesthesiologists

**Table 2:** Surgical Outcomes.

VARIABLES		RESULTS
Total operative time (mean±SD), min		345±66.9
Time of LRC and PLND / (mean±SD), min		106±22.0
Time of ICNB (mean±SD), min		204±46.4
EBL (mean±SD), mL		192±146
Transfusion, n (%)		0
ICU after surgery, n (%)		0
POD 1 VAS score (median [range])		1 (0-4)
Time of intake of liquid diet / (median [range]), day		4 (3-12)
Time of ambulation (median [range]), day		1 (1-4)
Length of hospital stay / (median [range]), day		14 (8-22)
**30-day complication rates, n (%)**		
Minor (I-II)		4 (19.0%)
Major (III-V)		0
**30-90-day complication rates, n (%)**		
	Minor (I-II)	3 (14.3%)
	Major (III-V)	1 (4.8%)
30-day readmission, n (%)		1 (4.8%)
Lymph node yield (mean±SD), n		18±9.2
Positive surgical margin, n (%)		0
Incidental prostate adenocarcinoma, n (%)		2 (10.5%)
Lymph node positive patients, n (%)		3 (14.3%)

**LRC**=laparoscopic radical cystectomy; **PLND**=pelvic lymph node dissection; **ICNB**=intracorporeal neobladder; **EBL**=estimated blood loss; **POD**=postoperative; **VAS**=Visual analogue scale

**Figure 3:**
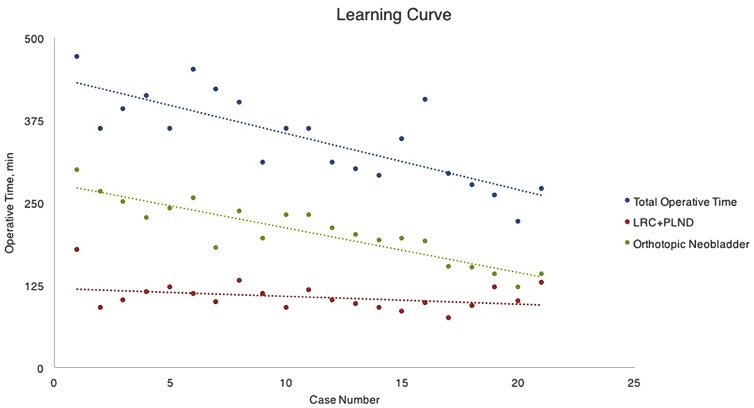
Learning Curve of the laparoscopic radical cystectomy with intracorporeal orthotopic neobladder.

The mean operative time was 345.6±66.9 min, including 106±22 min for LRC and PLND and 204±46.4 min for neobladder reconstruction. Figure-3 shows the learning curve of the procedure. The curve of LRC and PLND time was relatively stable, while the ICNB time gradually decreased. A significant decrease of mean ICNB times was observed in the first ten cases compared with the last ten cases (237.3 min vs. 169.0 min, P<0.001) (Table-3). The mean established blood loss was 192 mL without intraoperative transfusion, and no patient was sent to ICU after surgery. The median time of ambulation was POD 1 (range: 1-4). The median time of intake of liquid diet was 4 days (range: 3-12). The ureteric stents were removed on POD 14, and the catheter was removed on POD 16 after cystography confirmed no urine leaked from the bladder. The median length of hospital stay was 14 days (range: 8-22).

**Table 3:** Comparison of the first and last ten cases.

	Case (1-10)	Case (12-21)	P
Total operative time (mean±SD), min	393±47.6	297±50.0	<0.001
LRC and PLND time (mean±SD), min	114±25.9	97.7±15.8	0.11
ICNB time (mean±SD), min	237±33.7	169±31.3	<0.001
EBL (mean±SD), mL	155±95.6	240±182.3	0.21

**LRC**=laparoscopic radical cystectomy; **PLND**=pelvic lymph node dissection; **ICNB**=intracorporeal neobladder; **EBL**=estimated blood loss

Overall incidence of complications of any grade at 90-d was 33.3%, and major complications (Clavien grade ≥3) occurred in one patients (4.8%). One patient had prolonged ileus and urinary tract infection at one week postoperatively, who was treated with antibiotics (Clavien grade 2). One patient had ileus at 2 weeks postoperatively, which was resolved by supportive treatment (Clavien grade 1). One patient had urinary tract infection and was treated with antibiotics (Clavien grade 2). Three patients suffered urinary tract infection at 30-90 d and were treated with antibiotics (Clavien grade 2), one of them was readmitted for treatment of urinary tract infection. One patient had incision hernia at 8 weeks postoperatively and received hernia repair (Clavien grade 3b).

Pathologic results showed TisN0M0 for two patients, T1N0M0 for eight patients, T1N1M0 for one patient, T2N0M0 for six patients, T2N3M0 for one patient, T3N0M0 for one patient, T3N2M0 for one patient and small cell cancer for one patient. The median lymph node yield was 18 (range: 2-38), and 3 patients had positive lymph nodes (1/18, 2/24, 18/38). All surgical margins were negative. Incidental prostate cancer was detected in 10.5% (2/19) of the patients.

The median follow-up time was 15 (3-30) months. No patient suffered hydronephrosis access by ultrasound or abdominal CT. The continence and neobladder function outcomes are shown in Table-4. The complete daytime continence rate (pad-free) at 6 months and 12 months were 88.2% (15/17) and 85.7% (12/14), respectively. However, the complete night continence rate at 6 months and 12 months were 42.9% (6/14) and 57.1% (8/14), respectively. Four patients need one pad per night at 12 months. The neobladder capacity was 214 mL (170-330) and 375 mL (310-495) at 6 months and 12 months measured by ultrasound, respectively. One patient needed clean intermittent catheterization at 12 months follow-up. Only two patients received urodynamic study at 12 months, and the neobladder pressures were 12 cmH2O and 17cmH2O, respectively.

**Table 4:** Continence and Neobladder function Outcomes.

VARIABLES		6 MONTHS	12 MONTHS
**Urinary Continence**			
	Day time continence 0-1 pad/d	15/17 (88.2%)	12/14 (85.7%)
	Night time continence 0-1 pad/d	6/14 (42.9%)	8/14 (57.1%)
**Neobladder function**			
	Neobladder Capacity (Median [range]), mL	214 (170-330)	375 (310-495)
	Residual volume (Median [range]), mL	27 (0-135)	38 (20-160)
	Max flow rate (Median [range]), mL/sec	17 (9-22)	19 (8-21)
Clean intermittent catheterization		0	1

During follow-up period, no patient experienced recurrence. One patient died from myocardial infarction six months postoperatively, and two patients suffered lung metastasis at five months and six months postoperatively, whose TNM stage were T3N2M0 and T2N3M0, respectively.

**Figure 4:**
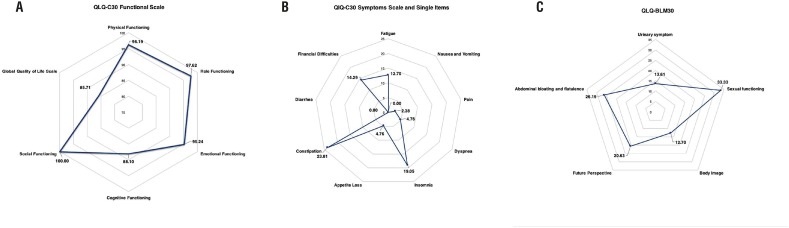
A) EORTC QLQ-C30 Functional Scale: a high scale score represents a high/healthy level of functioning (0-100). For the global quality of life scale, a high scale score represents a low level of functioning. B) EORTC QLQ-C30 Symptoms scale and single items: a high scale score represents a high level of symptomatology/problems (0-100). C) EORTC QLQ-BLM30: a high scale score represents a high level of symptomatology/problems (0-100).

Of 21 patients, only seven patients were followed up for more than one year and completed the QLQ-C30 and QLQ-BLM questionnaires. Mean questionnaire scores (QLQ-C30, QLQ-BLM30) are shown in the Figure-4. The lowest level of functioning in QLQ-C30 was “global quality of life” domain, and the highest level in QLQ-C30 was “social functioning” domain. The lowest level of QLQ-C30 symptoms scale was found in the “nausea and vomiting” and “diarrhea” domain and the highest level in the “constipation” domain. The QLQ-BLM30 questionnaire revealed the lowest level of symptomatology in the “abdominal bloating and flatulence” domain and the highest level in the “sexual functioning” domain.

## DISCUSSION

Laparoscopic and robotic techniques have been increasingly advocated for the potential advantages, such as decreased blood loss, decreased analgesic requirements and quicker recovery (12, 13). Some studies demonstrated that complete ICNB reconstruction may lead to decreased bowel exposure, reduction of insensitive fluid losses, and shorter time to oral intake (14). Recently, more and more studies reported robot-assisted ICNB reconstruction with acceptable operative time, complication rates and oncologic outcomes (7, 15, 16). However, the experience of laparoscopic ICNB reported in the literature is limited because of the complex and time consuming procedure (3, 8, 10). Compared to laparoscopic technique, robotic systems simplified suturing procedure which lead to a decrease in ICNB reconstruction time because of 3-dimensional vision and robotic arms. Although 3-dimensional laparoscopic technique could cover the shortage of vision, many surgical improvements are still required for traditional laparoscopic ICNB reconstruction.

In this study, we demonstrated a time efficient neobladder reconstruction and described many surgical improvements which could facilitate the procedure of ICNB without compromising complications. The time efficient neobladder has bilateral isoperistaltic afferent limbs which was reported by us before in the open approach (17). Having bilateral isoperistaltic afferent limbs, the left ureter could be anastomosed with left limb in situ avoiding being exceedingly mobilized. Additional mobilization of the left ureter for being brought to the right side could worsen the blood supply and thus lead to the development of ureteroileal stricture because of chronic ischemia (18). The surgical improvements are: (1) the division and continuity of the ileum was made using laparoscopic staplers; (2) “pulling technique” was applied during neobladder wall suture, which could facilitate suture and decrease ICNB time; (3) the ureters were end-to-end anastomosed with ipsilateral limb directly. In our experience, no patient developed ureteroileal stricture after the end-to-end anastomosis of ureter and ileal limb, and unidirectional peristalsis of the ureters and the afferent tubular ileal segment sufficiently protected the upper urinary tracts combined with low pressure neobladder. After applying the surgical improvements, the ICNB reconstruction time decreased from average 237 min for first ten cases to 169 min. for the last ten cases.

In many institutional centers worldwide, orthotopic neobladder has now replaced the ileal conduit as the standard form of reconstruction. Several types of neobladder have been described, but all should have the following features: high capacity, low pressure, absence of reflux, and complete voiding by abdominal straining and perineal relaxation (19). In our study, the neobladder capacity was about 375 mL, which was smaller than mean volumes reported for open neobladder construction of 450-524 cm3 (20,21). However, no patient has hydronephrosis or ureterectasis one year after operation. Two patient's neobladder pressure was low (12 cm H2O and 17 cm H2O). We will ask more patients to receive urodynamic study to evaluate the function of the neobladder. The most common postoperative complication is urinary tract infection, which can be well controlled by antibiotics.

The EORTC QLQ-30 and the QLQ-BLM30 module are the most commonly used generic and disease-specific instruments. Comparing to a study by Ciro Imbimbo et al., mean values obtained from the physical functioning, role functioning, emotional functioning, social functioning and global quality of life scale were higher in the present study (22). Cognitive functioning value was lower in the present study because one patient suffered memory difficult at one year after operation. The symptom scales of fatigue, nausea and vomiting, pain, dyspnea, appetite loss, diarrhea and financial difficulties were better than the results reported in the study by Ciro Imbimbo et al. (22). Our results have demonstrated lower mean values for the urinary symptom, body image and future perspective and higher mean values of sexual functioning and abdominal bloating and flatulence compared with published data. Our data showed that sexual functioning was worse because only one of the seven patients underwent nerve-sparing surgery.

This study has several limitations. Firstly, this is a retrospective study with small sample size and short length of follow-up. Secondly, the orthotopic neobladder is based on our experience and practice, and more urodynamic and longtime follow-up data are required to evaluate its function.

## CONCLUSIONS

We described our experience of 3D LRC with a novel intracorporeal orthotopic ileal neobladder, and many technique improvements facilitate the procedure. However, further studies are required to evaluate long-term outcomes of the intracorporeal neobladder with bilateral isoperistaltic afferent limbs.

## ABBREVIATIONS

RC: =Radical cystectomy
PLND: =Pelvic lymph node dissection
ICNB: =Intracorporeal neobladder
LRC: =Laparoscopic radical cystectomy
